#  Antibiotic resistance status and its costs in hematological patients: A two-year analysis

**DOI:** 10.22088/cjim.8.4.276

**Published:** 2017

**Authors:** Habip Gedik

**Affiliations:** 1 Department of Infectious Diseases and Clinical MicrobiologyMinistry of Health Okmeydanı Training and Research Hospitalstanbul, Turkey

**Keywords:** Hematologic neoplasms, Febrile neutropenia, Cost analysis, Anti-bacterial agents, Drug resistance.

## Abstract

**Background:**

Most frequently, empirical antibiotic therapy is immediately administered for patients with febrile neutropenia (FN). In this study, its aim was to assess the antibiotic resistance status and the cost of antibacterial agents in FN patients associated with hematological malignancies.

**Methods:**

The cost of antibacterial agents used in FN episodes in patients with hematologic neoplasms followed-up at the Department of Hematology from November 2010 to November 2012 were analyzed retrospectively.

**Results:**

In the study period, 15 of 141 patients who were admitted to the hematology ward and ineligible for the study criteria were excluded. In total, 282 febrile episodes of 126 consecutive patients with neutropenia were retrospectively investigated. Imipenem was found to be the most commonly used among the antibacterial drugs as 1.16 patient daily dose (PDD)/100 patient-days, 117.16 is the mean defined daily dose (DDD) per month and US $73264.66 total cost per year, followed by meropenem, cefoperazone-sulbactam, and linezolid.

**Conclusion:**

Choosing noncarbapenem-based antibacterial therapy for empirical treatment of FN until the growth of microorganisms and switching to carbapenem therapy subsequent to new radiological, or microbiological, or/ and clinical findings, the appropriate vancomycin use may decrease the cost of antibacterial agents in the treatment of FN episodes in patients with hematologic malignancies contributing to antimicrobial stewardship.

**F**ebrile neutropenia (FN) commonly develops secondary to chemotherapy in the patients with hematologic neoplasms. FN was described as an oral temperature > 38.3 °C or two consecutive fever measurement > 38.0 °C for two hours with a complete neutrophil count < 0.5×10^9^/L, or expected to be below 0.5×10^9^/L (1-3). Bacteremia and invasive fungal infections cause mortality apart from complications related to hematologic maligncies. Antibacterial and antifungal agents are usually administered in the treatment of febrile neutropenic episodes. Empirical antibiotic therapy is immediately administered for the survival of the patients with febrile neutropenia (1-3). Prolonged neutropenia and chemotherapy-induced mucositis cause gram-negative bacteremia in patients with hematologic malignancies. Unfortunately, the global spread of infections caused by multidrug-resistant (MDR) Gram-negative bacteria is increasing, and empirical antibiotic coverage may not adequately treat these bacteria (4). Fecal colonization with extended-spectrum beta lactamase-producing *E. Coli *is associated with increased risk of bloodstream infections (BSI) and higher cost with an average of $6528±$4348 (5). The aim of this study was to assess the antibiotic resistance status and the cost of antibacterial agents in patients with FN associated with hematologic malignancies.

## Methods

The cost of antibacterial and antifungal agents used in the treatment of FN episodes in patients aged 14 years and more with hematologic malignancies followed-up at the Department of Hematology of the Ministry of Health Okmeydanı Training and Research Hospital, an 800-bed tertiary hospital in İstanbul, Turkey between November 2010 and November 2012 were analyzed retrospectively. The hospital ethics committee has approved and registered this study (Date: 29.12.2012, Number: 51). The patients’ records were evaluated retrospectively if the patient had developed at least one neutropenic episode secondary to chemotherapy in the hematology ward. 

The patients who had one or any other hematological diseases (anemia, idiopathic or immune thrombocytopenic purpura, etc.) were not evaluated further. The hematology ward includes 23 beds, 1 single, 3 double, and 4 quadruple rooms without high-efficiency particulate air (HEPA) filters. The patients and their attendants were residing in the same room and using three toilets. A nurse and a doctor gave a weekly 1-hour instructional program about drug-resistant microorganisms and preventative measures to patients and their attendants while residing in the hematology ward. 

FN is the development of fever with oral temperature >38.3 °C or two consecutive fever measurements > 38.0 °C for two hours with a complete neutrophil count <0.5×10^9^/L, or expected to be below 0.5×10^9^/L (1). The patients’ data including demographics, hematological diagnosis, time and duration of febrile neutropenia episodes, clinical, radiological and laboratory findings, antimicrobial treatments, and outcomes were taken from hospital medical archives. 

The management of FN was implemented according to the clinical practice guidelines of both the Infectious Diseases Society of America (IDSA) and the European Organization for Research and Treatment of Cancer/Invasive Fungal Infections Cooperative Group and the National Institute of Allergy and Infectious Diseases Mycoses Study Group (EORTC/MSG) (1-3). Multinational Association for Supportive Care in Cancer (MASCC) score was used for risk evaluation of complications (1).

The therapeutic regimens which are active against *Pseudomonas aeruginosa, *were piperacillin-tazobactam (PIP-TAZ), cefoperazone-sulbactam (CEP-SUL) and PIP-TAZ in combination with ciprofloxacin (CIP), in accordance with local antibiotic resistance status as fist line antibacterial therapy. The antibacterial treatment was changed if the patient had new clinical, microbiological, or radiological findings after two days of empirical antibiotic therapy. Vancomycin was added to the treatment based on the aforementioned guidelines. 

The patients with vancomycin-resistant enterococcal (VRE) bacteremia were treated with linezolid (2x600 mg/day) for at least 14 days. While those patients with vancomycin-sensitive enterococcal (VSE) bacteremia were treated with ampicillin-sulbactam (8-12 gram/day) plus gentamycin (160-240 mg/day) for at least 14 days. Furthermost, the participants who had bacteremia caused by carbapenem-resistant gram-negative bacteria (CR-GNB) were treated with colimycin as a monotherapy or combined with other susceptible antibiotic for at least 14 days. The recommended dose of this drug is 2.5 to 5 mg/kg of colistin base activity per is equivalent to 6 to 12 mg/kg of colistimethate sodium per day. 

Daily doses of antimicrobial drugs were adjusted if the patient experienced liver or renal failure. Antifungal and antiviral treatments were not considered in this study as well as antibiotic prophylaxis was not administered to patients.

The defined daily dose is the presumed average maintenance dose per day of a drug used for its main indication in adults. DDD should reveal the global dosage, regardless of genetic changeability of drug metabolism. The essential principle is to allocate only one DDD per route of administration within an Anatomical Therapeutic Chemical (ATC) code. 

A DDD will only be allocated for drugs that has an ATC code. Meropenem (3 gr, J01DH02), vancomycin (2 gr, J01XA01), imipenem + cilastatin sodium (2 gr, J01DH51), teicoplanin (0.4 gr, J01XA02), colistin (3 MU, J01XB01), linezolid (1.2 gr, J01XX08), daptomycin (0.28 gr, J01XX09), piperacillin-tazobactam (14 gr, J01CR05), cefoperazone combinations (4 gr, J01DD62), sulfamethoxazole (80 mg) and trimethoprim (16 mg)  powder for injectable (20 UD= 20 ml), J01EE01) were evaluated to calculate the cost according to WHO Collaborating Center for Drug Statistics Methodology (6). Density of antifungal drug use was analyzed as yearly patient daily dose (PDD)/100 patient days. 

The costs of antimicrobial drugs were calculated by converting the price that was billed to the Republic of Turkey Social Insurance Institution per patient using United States dollar **($)** exchange rate. Quinolones were not assessed in this study since they were administered less than other antibacterial drugs and constitute a lower cost. 

Continuous variables were revealed as the mean ± standard deviation and range. Percentile values were revealed with two decimals. The overall 30-day crude mortality rate was calculated as the number of deaths within 30 days of neutropenia divided by the number of all patients. The infection-related mortality rate was calculated as the number of patients who died of infection during the neutropenic episodes divided by the number of all patients. 

## Results

In the study period, 15 of 141 patients were admitted to the hematology ward and ineligible for the study criteria were excluded. In total, 282 febrile episodes of 126 consecutive patients with neutropenia were retrospectively investigated. The mean age was 51.73±14.4 years (range: 17–82 years) and 66 were male patients. The mean MASCC score was 17.18±8.27, and the mean duration of neutropenia was 29.38±6.95 days. 

Of the 282 febrile episodes in 126 patients, 66 (23%) episodes of bacteremia and 24 (8%) episodes of fungemia were documented in 48 (38%) and 18 (14%) patients, respectively. Distribution of hematological malignancies of the patients was presented in table 1**. **

**Table 1:** Distribution of hematologic neoplasms in the patients

**Hematologic malignancies**	**n (%)**
Acute myeloblastic leukemia	73 (58)
Acute lymphocytic leukemia	22 (17)
Non-Hodgkin lymphoma	7 (5)
Chronic lymphocytic leukemia	5 (4)
Multiple myeloma	5 (4)
Hairy cell leukemia	4 (3)
Aplastic anemia	3 (2)
Chronic myeloid leukemia	2 (2)
Plasma cell leukemia	2 (2)
Mantle-cell lymphoma	2 (2)
Chronic lymphocytic leukemia with Burkitt's lymphoma	1 (1)
Total	126 (100)

Gram-negative bacteria (GNB) caused 74% of all bacteremia episodes. The etended – spectrum beta-lactamase (ESBL) E.coli is the most common organism causing Gram-negative bacteria bacteremia (21%), followed by ESBL (-) *K.pneumoniae* (12%), vancomycin-sensitive *Enterococcus faecalis* (9%), carbapenem-sensitive *P.aeruginosa* (9%), ESBL (+) *K.pneumoniae *(8%), and carbapenem-resistant *Acinetobacter baumannii* (6%). CR-GNB (6) caused 12% and 9% of Gram-negative bacteremia episodes and all bacteremia episodes, respectively. 

Clinical and microbiological responses were accomplished administering either PIP-TAZ or CEP-SUL therapy in 76% (32/42) of the cases with bacteremia caused by carbapenem-sensitive gram-negative bacteria (CS-GNB). The fatality rates were 50% in six patients with bacteremia caused by CR-GNB, while two death cases were associated with carbapenem-resistant *A. baumannii *and the other with carbapenem-resistant *P. aeruginosa*. Colistin monotherapy was administered to two patients with CR- *A. baumannii* and one patient with CR-* Serratia marcescens, *while another patient who received colistin monotherapy died. Colistin and rifampicin combination was administered to a patient with CR- *A. baumannii *and another patient with CR-*P.aeruginosa*. Colistin and sulbactam-cefoperazone combination was administered to a patient with CR- *A. baumannii *who died eventually*. *

The hematological malignancies of those patients were acute myeloid leukemia (AML) in three cases, non-Hodgkin's lymphoma (NHL) in one case, multiple myeloma (MM) and acute lymphoblastic leukemia (ALL) in other cases. 

Imipenem was found to be the most commonly used antibacterial drugs as 1.16 PDD/100 patient- days, with 117.16 mean DDD per month, $73264.66 total cost per year, followed by meropenem, cefoperazone-sulbactam, linezolid. Total cost per year was calculated as $261,156.38 for antibacterial drugs. Total expenditure per patient was detailed as $3704.34 for antibacterial drugs, likewise, the costs of all antibacterial drugs per FN episode were $1852.17 (table 2). 

Overall the 30-day crude mortality rates were 31% of patients diagnosed with AML, acute lymphocytic leukemia, MM, chronic myeloid leukemia, NHL Infection-related mortality was calculated as 22%.

**Table 2:** The costs by antibacterial agents

**Antibacterial drug**	**DDD**	**PDD/1000 patient days**	**Mean DDD per month**	**Range of DDD per month**	**Mean cost per month ($)**	**Range of cost ($) per month**	**Total cost per year ($)**
Sulfamethoxazole/ Trimethoprim	20 UD (= 20 ml)	0.039	275.25	0- 285,5	105.62	0-185.11	1131.55
Colistin	3 MU	0.001	4.19	0-21	40.40	0-910.53	5587.86
Piperacillin-tazobactam	14 gr	0.007	11.28	0-49	482.56	0-2087	6032.09
Teicoplanin	0.4 gr	0.007	15.48	0-65	447.02	0-1886.81	11175.74
Vancomycin	2 gr	0.021	44.2	0-128	1015.66	0-2986.08	12695.9
Linezolid	1.2 gr	0.007	4.2	0-58	1072.1	0-3876.74	13401.5
Cefoperazone-sulbactam	4 gr	0.038	78.48	10-142	3826.25	1021.14-5662.7	39527.51
Meropenem	3 gr	0.029	52.74	0-150	3313.06	0-10570.84	48039.57
Imipenem	2 gr	0.116	117.16	15-220	5861.17	828.18-24293.41	73264.66
Total							261,156.38

DDD = defined daily dose; PDD = patient daily dose.

## Discussion

Gram-positive bacteria predominately caused bloodstream infections in patients with neutropenia after the 1960s and 1970s (7). Gram-negative bacteria came into prominence in the bacteremia attacks of hematological patients (8). Carbapenem-resistance rates (23%) in the Gram-negative isolates were notably higher than ESBL production rates (15%) in our hematology ward. Carbapenems cause the selection of carbapenem-resistant Gram-negative bacteria during the antibacterial treatment of hematological patients (9). Carbapenems are usually initiated and associated with notable high cost due to the infections frequently caused by Gram-negative bacteria during the febrile neutropenia episodes. Non-carbapenem- based therapy was effective in two thirds of infections caused by Gram-negative bacteria. Cefoperazone-sulbactam was firstly initiated as a non-carbapenem based therapy. In case there is a deterioration of clinical findings or growth in culture with persistent fever, switching to antibiotics is recommended to decrease cost and antimicrobial resistance (1-3). Piperacillin/tazobactam was used less than cefoperazone/sulbactam, thus its cost was less than the cefoperazone/sulbactam. Nonetheless, piperacillin/ tazobactam is used four times per day as 4x4.5 gr/iv, and cefoperazone/sulbactam is used three times daily as 3x2 gr/iv. Hence cefoperazone/sulbactam provided time-saving for healthcare staff. Imipenem has been administered more than meropenem since it is cheaper. Drug-drug interactions should be taken into consideration besides the selection of antimicrobials. Carbapenem use was likely to increase carbapenem-resistant gram-negative bacteria colonization and related infections except that it increased the cost in our study (10). Persistent fever was reported to be the common reason for switching to carbapenem-based therapy (10). Patients with fever under non-carbapenem based therapy, and did not yield any microorganisms in the microbiological cultures, were related to the increased carpapenem-use in our study. Though drug interactions and elevated liver enzymes could contribute to more imipenem use, there was no any other specific reason for the choice. Colistin was administered to six patients with carbapenem-resistant gram-negative bacterial infections either as monotherapy or combination therapy with a 50% mortality rate. Yet, the colistin-related cost remained lower than the cost of other antibacterial agents, notwithstanding, the infections caused by carbapenem-resistant gram-negative bacteria increased total cost of hospital care. Sulfamethoxazole/trimethoprim, which was the least expensive antibiotic in our study, was administered for probable opportunistic infections. On the other hand, linezolid was administered less than vancomycin due to its side effects. At any rate, linezolid treatment was more cost-effective than vancomycin. 

Gram-positive bacteremia comprised one quarter of all bacteremia cases (table 2). Bacteremia due to methicillin resistant staphylococci occur and vancomycin-resistant enterococci (VRE) and colonization with VRE are the main causes of total cost of antibacterial drugs that are active against Gram-positive bacteria in our study. Vancomycin, linezolid, and sulfamethoxazole/trimethoprim were substantially administered more than teicoplanin for the treatment of pneumonia. Vancomycin has more cost-effectiveness than linezolid and teicoplanin for the treatment of infections caused by gram-positive bacteria, in case there are no contraindications. Although linezolid was used less than glycopeptide antibiotics, its cost was more expensive. Linezolid should be opted for the patients with infections caused by VRE or pneumonia originated from resistant Gram-positive bacteria, or renal failure. Limited use of linezolid and glycopeptide antibiotics via guidelines may reduce the costs related to unnecessarily broad-spectrum antibiotic use.

When the number of FN episodes is prolonged 30 days on average together with the number of patients in our study to be taken into account, these costs are definitely predicted. In the study of Lathia et al., antibacterial-related costs were reported $520±587 (range: $3010-$21,847) per FN episode for the patients who were in MASCC high-risk group and had a length of hospital stay of 8.8±5.9 days (11). Besides, the total costs were reported to be ranging from $908 to $2543 greatly among the seven regimens for 14 days of therapy (12). The direct medical cost of treatment of a FN episode in the hospital was estimated between $ 2,000 to $ 11,000. Hospital room was reported to constitute the most expenditure (58-78%) in the distribution of cost components, followed by antibiotics (19-26%) and diagnostic tests (3-16%) in the treatment of FN in Canada and the United Kingdom (13). The use of antibiotics and their cost is similar to those reported with such antibiotic cost, incurred in our study. 

In conclusion, choosing the non-carbapenem based antibacterial therapy for the empirical treatment of FN until the growth of microorganisms, switching to carbapenem therapy subsequent to new radiological, or microbiological, or/ and clinical findings, and the limited vancomycin use may decrease the costs of antibacterial agents in the treatment of FN episodes of patients with hematologic neoplasms contributing to antimicrobial stewardship.

## Funding:

Nil

## Conflicts of Interest:

None declared.
